# Development of an individual index of social vulnerability that predicts negative healthcare events: a proposed tool to address healthcare equity in primary care research and practice

**DOI:** 10.1186/s12939-023-01965-9

**Published:** 2023-08-18

**Authors:** Jeannie Haggerty, Simona C. Minotti, Fatima Bouharaoui

**Affiliations:** 1 McGill University, Department of Family Medicine, Montréal, Québec H3S 1Z1 Canada; 2 St. Mary’s Hospital Research Center, Hayes Pavilion – S.4720, 3830 Lacombe Ave., Montréal, Québec H3T 1M5 Canada; 3 Institute for Better Health, Trillium Health Partners, 100 Queensway W, Mississauga, ON L58 1B8 Canada; 4 Department of Statistics and Quantitative Methods, University of Milano-Bococca, Milano, Italy

**Keywords:** Primary health care, Vulnerable populations, Healthcare disparities, Social determinants of health psychometrics

## Abstract

**Purpose:**

Socially disadvantaged patients may lack self-efficacy to navigate a complex health system making them vulnerable to healthcare inequity. We aimed to develop an Index of social vulnerability that predicts increased risk of negative healthcare events (e.g. emergency hospitalization), independent of chronic disease burden. The analysis illustrates the conceptual and practical steps leading to the development of a pragmatic Index of social vulnerability to limited healthcare self-efficacy.

**Methods:**

Using data from a 3-year cohort of 2507 adult primary care patients in Québec (Canada), we applied two complementary structural equation modelling approaches—Partial Least Squares Path Modelling (PLS-PM) and Multiple indicators and Multiple Causes (MIMIC) modelling—to identify a minimal set of social characteristics that could be summed into an Index related to limited healthcare self-efficacy. We then used logistic regression to determine if the Index predicted: hospital emergency department use; hospital admissions; unmet need for care, and others. We privileged parsimony over explanatory capacity in our analytic decisions to make the Index pragmatic for epidemiologic and clinical use.

**Results:**

The Individual Social Vulnerability Index is the weighted sum of five indicators: two indicators of social support; educational achievement; financial status; limited language proficiency. The Index predicts increased likelihood of all negative healthcare outcomes except unmet need, with a clear threshold at Index ≥ 2. The effect is independent of chronic disease burden.

**Conclusion:**

When social deficits outweigh social assets by two or more (Index ≥ 2), there is an increased risk of negative healthcare events beyond the risk attributable to poor health. The Index is a pragmatic tool to identify a minority of patients who will require additional support to receive equitable healthcare.

## Introduction

An equitablehealth system not only guarantees equal opportunity for healthcare but also actively facilitates access for those with greater needs or more barriers than most people. There is abundant evidence that social characteristics and circumstance drive health status and health need [1]. Our interest in this article is on equity of healthcare in response to that need because there is ample evidence of an inverse care law [2], where those at greater need receive less accessible and less appropriate healthcare despite their higher need [3, 4]. These individuals will have a higher rate of negative healthcare events such as use of the hospital emergency department for minor issues, emergency hospitalizations for some chronic and episodic conditions, and unmet needs for care [5, 6]. Healthcare inequity occurs when access to healthcare differs by non-health characteristics such as socioeconomic status rather than by health need [7]. We used access in a broadest sense that extends beyond mere use of services to include dimensions such as acceptability, accommodation, and appropriate care [8].

Healthcare equity is typically assessed retrospectively, by examining indicators of access stratified by different social characteristics [7, 9, 10]. In recent years, various initiatives have introduced more nuanced proposals for stratification in research that go beyond single social characteristics to capture the complexity of the various social characteristics that can make persons vulnerable to healthcare inequities [11]. There has been a parallel push for gathering social drivers of health in clinical practice [12, 13]. This article reports on an approach to identify a threshold at which social indicators suggest that the patient is vulnerable to negative healthcare events, and would benefit from more robust supports.

This inquiry began in the context of a research program proposing a primary healthcare performance measurement and reporting system for Canada [14], with healthcare equity as a key metric of high performance. We were particularly interested in identifying “complex vulnerable” patients whose combination of social and health challenges make them vulnerable to poor care experience and negative healthcare events. We aimed to identify a set of individual social characteristics that are associated with a higher likelihood of avoidable emergency department (ED) use, hospital admission, health status decline and/or unmet need for healthcare compared to non-vulnerable persons in similar health. Our goal was to combine the social characteristics into an Index that could be used as a stratifier to assess retrospectively potential healthcare inequity in a primary care performance system, but also that could be used proactively to identify patients at risk to provide them with greater support to avoid negative healthcare events.

In this article, we tell the research story of our development of an Individual Social Vulnerability Index that is related to limited healthcare self-efficacy and also predicts the risk of negative healthcare events. The research story starts with the description of an empirical case that seemed well-suited to secondary analysis for this purpose, that was followed by frustrated analytic attempts that sent us back to the drawing board (literally) to map a conceptual model that led to a fruitful collaboration and more appropriate analytic approach. We present this work to demonstrate the approach and results that led an Individual Social Vulnerability Index that we believe to be pragmatic and generalizable for reporting on health system equity and for detecting patients in clinical practice who need more robust support in obtaining appropriate care because they are a greater risk of negative healthcare events.

## Method

### Empirical case

The data came from a 3-year cohort of 2507 primary care users aged 25-to-75 years, selected in 2010 in urban, rural and remote areas of Quebec, Canada. The original cohort study explored how primary health care and patient characteristics interacted in determining healthcare trajectories, experiences and outcomes [15]. The data consisted of three annual patient surveys and administrative data for medical services and hospitalizations. One part of the cohort was recruited by random digit dialling (community cohort, *n* = 2406) and the other from the waiting rooms of 12 PHC clinics (clinic cohort, *n* = 1029). The response rate to the baseline survey (T1) was 72.9% (2507/3438), and cohort retention over three years was 80.9% (*n* = 2029). Seventy-one percent (1769/2507) consented access to medical services billings from the Quebec universal insurance agency, the *Régie de l’assurance-maladie du Québec,* used to ascertain use of the hospital ED and all hospitalizations.

The data was considered relevant to development of a social vulnerability index because a large number of social and healthcare factors were elicited at baseline, and outcomes on healthcare events and experience in the subsequent 2 years.

The social dimensions elicited were: age; sex; residential location; languages spoken at home; self-reported educational status; self-perceived financial status; occupation; household income; indicators of wealth (home and care ownership; retirement savings plan); social support. The annual questionnaire also elicited and indicators of healthcare self-efficacy, chronic illness burden using the Disease Burden Morbidity Assessment [16, 17], mental and physical functional health status (SF12^17^), self-reported ED use and reasons, unmet needs for healthcare and various dimensions of primary care experience.

### Initial frustration and clarity from mapping a conceptual model

A group of health services researchers (see acknowledgments) initially used logistic regression models to predict the likelihood of negative healthcare events as a function of social characteristics, controlling for health status. These attempts to identify a set of relevant social characteristics defied interpretation. Since social characteristics are associated with health status, we could not differentiate risk attribution of negative healthcare events due to social characteristics from the risk due to health status.

**Fig. 1:**
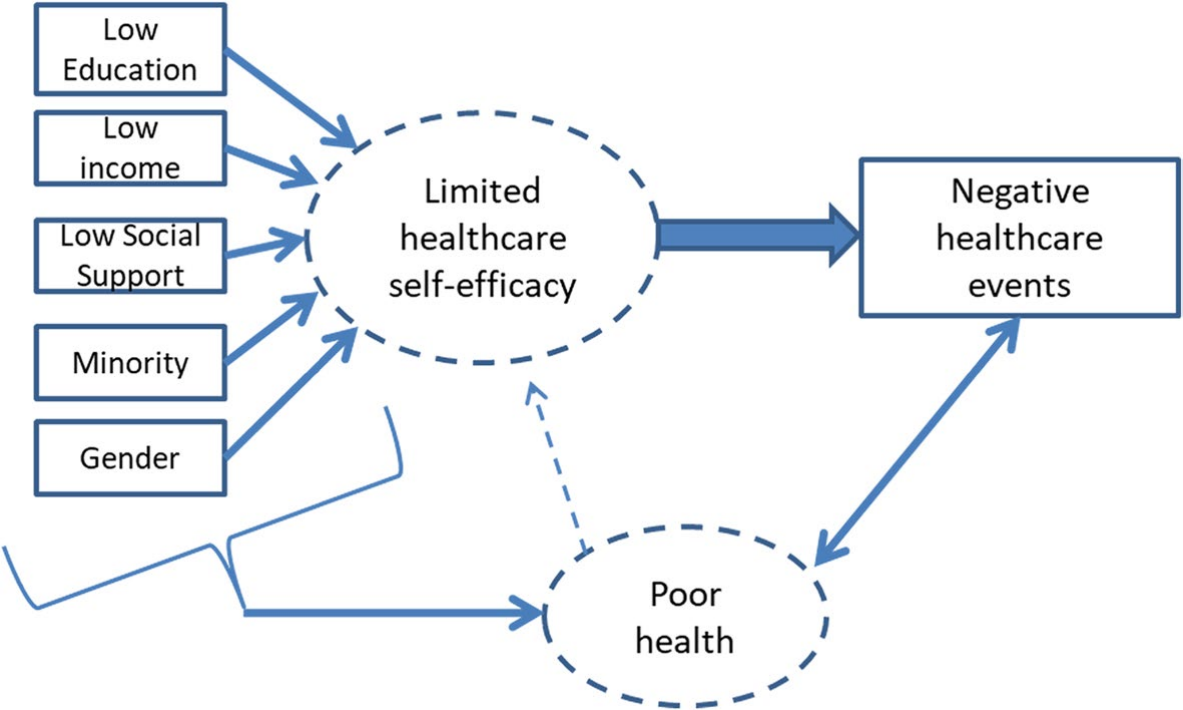
Mapping of concepts linking social vulnerability to poor healthcare: social factors cause both limited self-efficacy and poor health which lead to negative healthcare events and experience

This led us to map the concepts that explain the confounding and illustrate our hypotheses, Fig. 1. We posited that social characteristics such as low income, low educational attainment, and limited language proficiency compromised self-advocacy and self-efficacy in a complex and often-fragmented health system, and that a poor health state (even if temporary) could further increase vulnerability leading to negative healthcare events. We also recognize that the same social characteristics are conditions for poor health that may interact with healthcare self-efficacy to further increase the risk of negative healthcare events and experience. But low healthcare self-efficacy itself is hypothesized to be largely independent of health status in predicting negative healthcare events and experience. Indeed, the bivariate analysis showed that one variable in our dataset, self-efficacy in finding health information, was associated both with social characteristics and likelihood of some negative healthcare events and experience but weakly related to health status. The statisticians (SM, FB) saw in the conceptual logic that latent variable analysis is the most appropriate statistical approach to explore these effects and develop an appropriate index.

### Latent variable analysis

Latent variables (or constructs) are phenomena that are not measurable directly but can be approximated from observed indicators. Functional health and socioeconomic status are examples. Both Principal Component Analysis (PCA) and Factor Analysis (FA) are commonly-used to infer *latent variables* in social and health research. PCA and FA are frequently confused and referred to interchangeably, but as noted in the following brief overview of both PCA and FA, each has different underlying statistical assumptions and fundamentally different representations of the latent constructs. This will also serve to illustrate why these approaches are not entirely appropriate for our conceptual model.

### A non-technical primer: PCA vs. FA

**Fig. 2:**
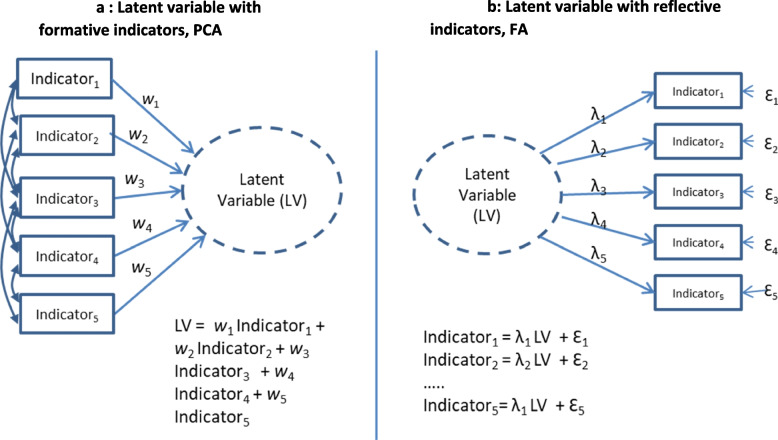
Representations of a latent variable in Principal Components Analysis (PCA) and Factor Analysis (FA). **a** Latent variable with formative indicators, PCA. **b** Latent variable with reflective indicators, FA

It is common practice to use PCA for “exploratory factor analysis” followed by “confirmatory FA” to posit measures. PCA is an exploratory technique with few formal statistical assumptions, usually used to reduce the number of variables that represent some common underlying phenomena. FA is a maximum likelihood statistical model that is used commonly to validate instruments. It produces comparative goodness-of-fit statistics for different indicator-construct configurations. Figure 2 depicts the differences between PCA (Fig. 2a) and FA (Fig. 2b). Below, we will draw attention to differences in the direction of the arrows between the indicators and the latent variable, the relationship between indicators, and the statistical representation of latent variable.

In a PCA model (Fig. 2a), the latent variable is conceived as being *caused by* the observed indicators, which are called *formative or causal indicators*(arrows emerge from indicators to latent variable). Formative indicators in PCA “need not be internally consistent nor exhibit a pattern of high positive correlation” [18]. Indeed, high inter-indicator correlation is not desirable; what matters is that each indicator makes a unique contribution to the latent variable after adjusting for the other indicators. Such a latent variable is conceived as *multi*dimensional. Statistically, the PCA latent variable is a linear combination (weighted sum) of the indicators with no error term. PCA is the approach that has been most used for multidimensional constructs such as socioeconomic status and material or social deprivation [19–23].

In a FA model (Fig. 2b), the latent variable is *assumed to cause* the observed indicators (arrows emerge from latent variable to indicators), so indicators are called *reflective indicators*. Each indicator is assumed to be a plausible parallel test of the construct, and indicators *must* be highly correlated. The latent variable is presumed to be *uni*dimensional. Statistically, with this maximum-likelihood covariance-based method, each indicator is a linear function of the latent variable, plus an error term. FA has been used to identify indicators for unidimensional constructs such as health literacy(ref) and self-efficacy(ref).

Neither PCA alone nor FA alone were appropriate for our conceptual model, which posits that the multidimensional construct of social vulnerability is related to the likely unidimensional construct of limited healthcare self-efficacy. First, our conceptual model suggests a relationship between latent constructs. While both PCA and FA can produce more than one latent construct, no relationship is assumed among latent constructs. But we are interested in social vulnerability that is conditioned on the construct of healthcare self-efficacy. Second, it suggests both formative and reflective indicators. The social indicators to vulnerability are assumed to be causal or formative, suggesting a PCA-like approach. The construct of healthcare self-efficacy is reflective of indicators like health efficacy, health information agency, self-management between medical visits and self-management without medical help.

### Combining and extending PCA and FA

We combined the strengths of both PCA and FA in a way that is more coherent with our conceptual model, by using two alternative structural equation modelling (SEM) approaches: i) Partial Least Squares Path Modelling (PLS-PM) and ii) Multiple indicators and Multiple Causes (MIMIC) modelling. Both extend PCA and FA models and draw on their strengths. Here, we describe each briefly.

### Partial Least Squares Path Modelling (PLS-PM)

**Fig. 3:**
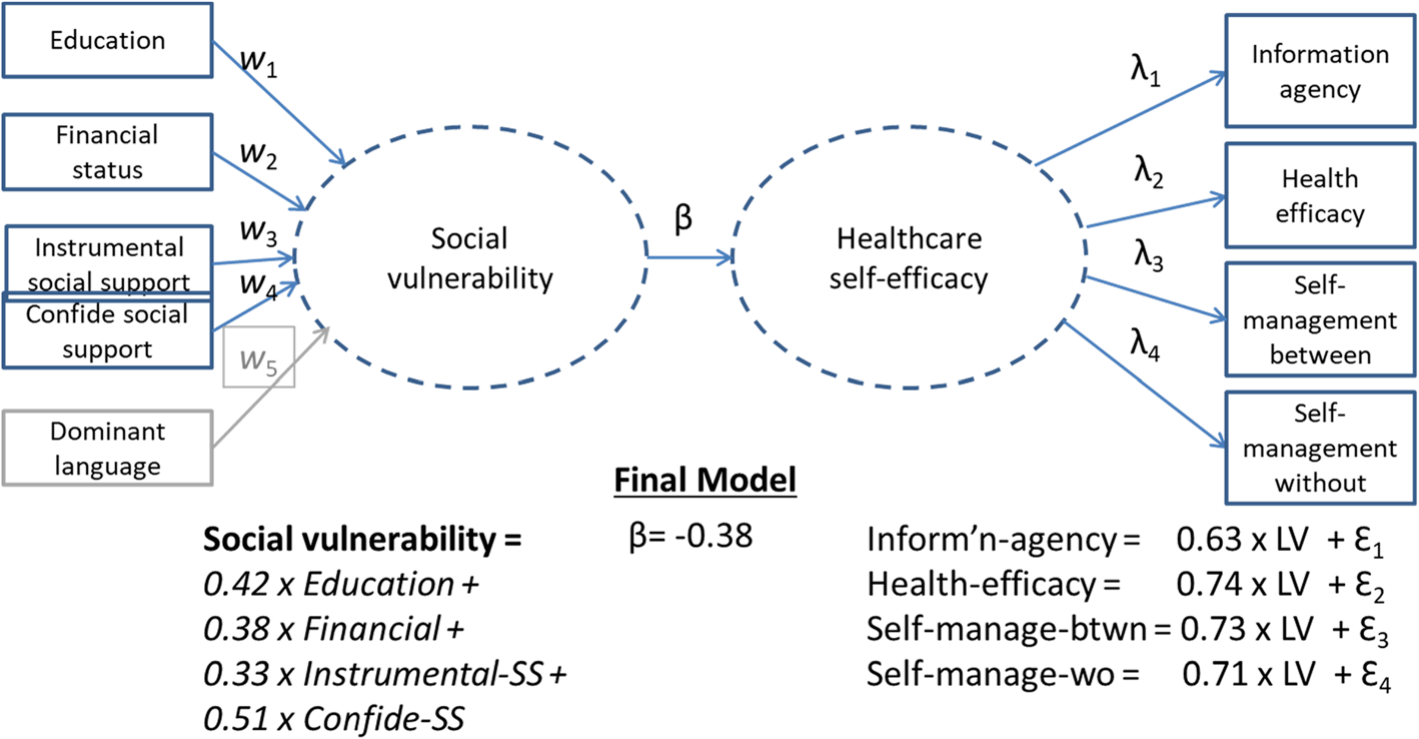
Illustration of PLS-Path Model, showing final model weights and loadings

This component-based SEM was introduced by Wold (1975) [24] under the name *nonlinear iterative partial least squares*. (Haenlein & Kaplan 2004 [25] provide an excellent nontechnical overview of PLS-PM). Like PCA, PLS-PM is recommended for exploratory phases because it does not make distributional assumptions about the variables and is not affected by small sample size [26, 27]. Represented in Fig. 3, the PLS- Path Model brings together the two related latent variables: 1)“social vulnerability”, caused by formative social indicators, and 2) “healthcare self-efficacy” reflecting relevant indicators. The strength of the relationship between the two latent variables is estimated by a path coefficient (beta).

The PLS-PM procedure estimates the multidimensional latent variable as an exact linear combination (weighted sum) of its formative indicators. The weights for the indicators (w_x_) and the loadings (lambda_x_) for the reflective indicators are estimated iteratively with the estimation of the latent constructs. The estimated weights for the formative indicators will differ from the pure PCA solution depicted in Fig. 2b, because the PLS-PM estimates maximize simultaneously the explained variance for the indicators and the correlation between latent variables as per the theoretical model [21]. An essential assumption in PLS-PM is that all the variance in the causal indicators are informative. This is tantamount to assuming that the indicators are completely relevant to the latent variable and are measured without error. A construct-level error term cannot be specified.

### Multiple indicators and Multiple Causes (MIMIC) modelling

**Fig. 4:**
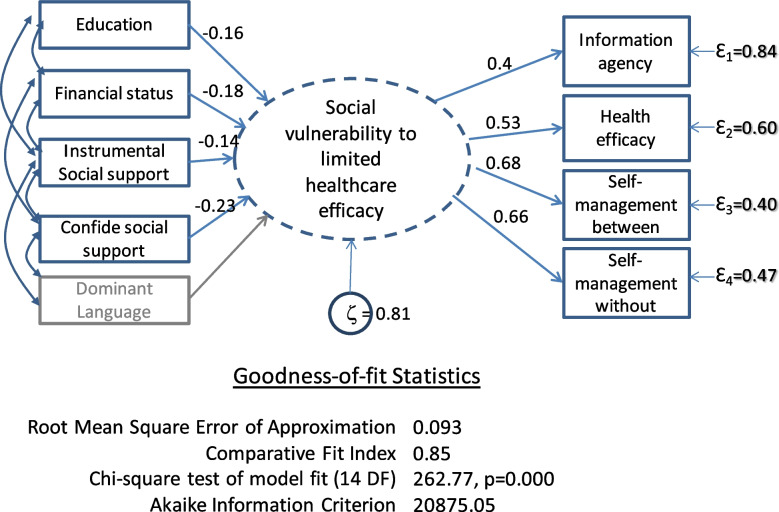
MIMIC Model Illustration and final results (model with disturbance or error term, ζ)

The second SEM approach, MIMIC modelling [28, 29], is a special case of maximum-likelihood covariance-based methods like confirmatory FA [30]. The MIMIC model differs from PLS-PM by specifying a single latent construct with both formative and at least two reflective indicators. Depicted in Fig. 4, the single latent variable of our conceptual MIMIC model is identified as “Social vulnerability to limited healthcare self-efficacy”, is caused simultaneously by inter-related formative indicators of social vulnerability and it reflects indicators of healthcare self-advocacy.

MIMIC has more restrictive statistical assumptions than PLS-PM, and requires that each formative indicator be normally distributed and have a similar proportional effect on each of the reflective indicators, that is, a similar strength of correlation [30]. This assumption can limit the indicators eligible for inclusion in the MIMIC model. Unlike PLS-PM, the MIMIC formative indicators are not assumed to explain completely the latent variable, and this is represented by an error or “disturbance” term (ζ) for the latent variable. MIMIC modelling generates goodness-of-fit statistics that permit comparative model testing.

For PLS-PM, we used R (PLS-PM package) to estimate the model, and for the MIMIC models we used three software packages to verify that results were similar despite different estimation algorithms. To specify the initial model, we used LISREL [31], which assumes that the indicators are continuous and normally distributed. We then estimated the model parameters using MPlus [32], which permits categorical variables, and finally R (lavaan package) for the final model estimates. It is possible to estimate a MIMIC model without a disturbance term, which we did to demonstrate that in this case MIMIC produced similar results to PLS-PM [2]. Our final model is specified with a disturbance term in recognition that the latent variable is measured with error.

## Results

Table 1 provides an overview of study population’s demographics, health and social characteristics. From our baseline sample 81.1% (2039/2507) had complete data. Those with complete data were less likely to be poor (χ2 = 8.77, 2df, *p* = 0.01) and less likely to have low educational attainment (χ2 = 8.77, 2df, *p* = 0.01).

**Table 1:** Demographic, social and health characteristics, showing differences between baseline population sample and analytic sample

**Characteristic**	**Total population at baseline (*n* = 2507)**	**Participants with complete data (n-2039)**	**Difference between samples**^a^
**Demographics**			
**Percent Women (n)**	62.0% (1555)	61.8% (1260)	**χ**^**2**^ = 0.25, 1df, *P* = 0.62
**Mean age (std. dev)**	51.9 years (12.6)	52.3 years (12.5)	t = -4.26, *p* < .0001
**Percent rural residents**	43.7% (1082)	44.3% (892)	**χ**^**2**^ = 1.36, 1df, *p* = 0.24
**Health**			
**Compared to others your age, how do you rate your health in general, % (n):**			**χ**^**2**^ = 6.35, 4 df, *p* = 0.17
Poor	3.98% (99)	3.78% (77)	
Fair	17.08% (425)	16.34% (333)	
Good	34.31% (854)	34.59% (705)	
Very good	31.50% (784)	31.80% (648)	
Excellent	13.14% (327)	13.49% (275)	
**Multimorbidity: Percent with 3 + chronic illnesses (list of 14)**	25.5% (636)	26.1% (530)	**χ**^**2**^ = 1.82, 1df, *p* = 0.18
**Social characteristics** (in order of increasing vulnerability)			
**Highest education completed, % (n)**			**χ**^**2**^ = 20.8, 2df, *p* < .0001
Primary or partial secondary	21.4% (531)	19.8% (403)	
Secondary or technical	55.1% (1366)	55.7% (1138)	
University	23.5% (582)	24.5% (500)	
**Self-perceived financial status, % (n)**			
Very poor to very tight	9.7% (238)	8,9% (181)	
Tight to Moderately comfortable	59.9% (1476)	60,1% (1225)	**χ**^**2**^ = 8.77, 2df, *p* = 0.01
Comfortable or very comfortable	30.5% (751)	31.0% (633)	
**Social Support—**How many persons, family or friends… **…could help you with activities of daily living (e.g. dressing, driving)?**			
More than one	50.9% (1262)	49.4% (1007)	χ^2^ = 13.14, 2 df, *p* = 0.001
One	31.6% (784)	33.1% (675)	
None	17.5% (434)	17.5% (357)	
**… can you freely confide in or talk about yourself or your problems?**			
More than one	73.3% (1822)	73.4% (1496)	
One	21.2% (526)	21.5% (438)	**χ**^**2**^ = 3.26, 2df, *p* = 0.20
None	5.5% (137)	5.1% (105)	
**Language spoken at home, % (n)**			
Other	1.2% (30)	0.9% (19)	**χ**^**2**^ = 16.95, 2 df, *p* = 0.0002
French or English	94.0% (2339)	94.9% (1933)	
French and English	4.7% (118)	4.1% (84)	

^a^Two-tailed statistical test of difference between baseline and between baseline sample and those included in the study: χ^2^ = value of chi-squared, with degrees of freedom; t = value of t-test

### Model specification

The four variables that we judged to best reflect the latent variable presumed to be healthcare self-efficacy are shown in Table 2, along with Spearman correlations between them. Each indicator has a median value of 4 but the full range 1-to-5 was present in the study sample. Correlations between all indicators are statistically significant, and medium strength between information agency and health efficacy and between the two self-management indicators.

**Table 2:** Spearman correlation within indicators reflective of limited healthcare self-efficacy, and between reflective and formative social indicators. (All reported correlations are statistically significant at *p* < 0.001)

**Indicator statement (Likert scale)**	Mean (SD)		Spearman Correlation		
		Inform’n efficacy	Health efficacy	Manage btn visits	Manage w/o help
1. **Health Information efficacy**: How easy is it for you to get healthcare information by yourself? (1 = not at all to 5 = Very easy)	3.78 (1.09)	1.0	0.40	0.20	0.22
2. **Health efficacy**: How much do you feel you have control over your health? (1 = None to 5 = Complete)	4.04 (0.92)			0.31	0.31
3. **Self-management between appointments**: How easy is it for you to care for your health condition between appointments? (1 = not at all easy to 5 = Very easy)	4.29 (0.87)				0.54
4. **Self-management without medical help**: In your day-to-day life, how do you rate your ability to take care of yourself without medical help? (1 = Very poor to 5 = Very good)	4.09 (0.92)				1.0
**Social (Formative) Indicators**^a^					
**Highest education completed** (1 = post-graduate university to 8 = primary)	**Mode** Secondary-technical (55%)	-0.17	-0.20	-0.13	-0.13
**Self-perceived financial status** (1 = very comfortable to 7 = very poor)	Moderate comfortable (60%)	-0.12	-0.18	-0.22	-0.17
**Social Support, instrumental activities** (0 = more than one person to 2 = no one)	High -1 + (49%)	-0.15	-0.12	-0.15	-0.15
**Social support, confidante** (0 = more than one person to 2 = no one)	High -1 + (73%)	-0.17	-0.19	-0.20	-0.18
**Language at home** 0 = Eng/Fr,1 = Other	Fr or Eng (94%)	–-	–-	–-	–-
		Inform’n efficacy	Health efficacy	Manage btn visits	Manage w/o help

^a^Social indicators in their original form, higher values reflect increased social vulnerability

Table 2 also shows the Spearman correlations between the indicators of limited healthcare efficacy and the candidate formative social indicators. Indicators of healthcare self-efficacy correlate negatively with low social support; poor financial status; and low educational achievement. The small to medium correlations are of similar magnitude, thus meeting the proportional effect assumption for MIMIC. We also examined correlations with age, sex, rurality and language, but they were very small or not statistically significant, so they were not included in the model. Nonetheless, we note that males, older adults, and rural residents tend to have lower values of healthcare self-efficacy indicators. A proxy of limited language proficiency – not speaking English or French at home – was not statistically significant and was not included in our model, because only 1% of the sample (*n* = 19/2039) expressed this vulnerability risk, so we did not have statistical power to reliably estimate the weight despite its conceptual relevance.

We undertook PLS-PM and MIMIC alternately, selecting the model that best represents our conceptual model but that also preserves the highest explained variance of the latent variable in PLS-PM and provides the highest CFI and lowest Akaike Information Criterion in MIMIC. After the final models were specified and tested, we transformed the social indicators into three ordinal categories, with cut-points informed by the highest explained variance in the latent variable with PLS-PM and the best comparative fit index (CFI) for MIMIC. The transformation not only improves model stability, but also has a common scoring (Table 3) that reflects the notion that a social characteristic can be a social asset protective against vulnerability (-1 = “not-vulnerable”) or a deficit leading to greater vulnerability (1 = “vulnerable”).

**Table 3:** Components of the individual index of social vulnerability, showing indicator values and distribution in the study population

**Indicator**	**Weights & Coding**	**Percent**^a^
**Highest education completed**	-1 = High (University completed or partial)	24.5%
	0 = Mid (Secondary completed or technical college)	55.7%
	1 = Low (None, primary or partial secondary)	19.8%
**Self perceived Financial status**	-1 = Comfortable or very comfortable	31.0%
	0 = Tight to moderately comfortable	60,1%
	1 = Very tight to Poor	8,9%
**Instrumental social support;** Number of family or friends to help with activities of daily living (e.g. dressing, driving)?	-1 = High social support (1 + persons)	49.4%
	0 = Medium social support (1 person)	33.1%
	1 = Low social support (0 persons)	17.5%
**Confidant social support** Number of family or friends you freely confide in or talk about yourself or your problems?	0 = high social support (1 + persons)	73.4%
	1 = Medium social support (1 person)	21.5%
	2 = Low social support (0 persons)	5.1%
**Index of social vulnerability to limited healthcare self-advocacy** (calculated by summing the value of the indicators)	-2 = Not vulnerable	22.2%
	-1 = mode, not vulnerable	26.1%
	0 = median, not vulnerable	22.6%
	1 = possibly vulnerable	15.1%
	2 = vulnerable	8.6%
	3 = very vulnerable	5.4%

^a^For simplicity we do not show 95% confidence intervals, but they vary by ± 1% where prevalence < 10% and by ± 2% where prevalence ≥ 10%

The weight and loading estimates of the final PLS-PM model are included in Fig. 3. The moderately strong path coefficient (β = -0.38) shows that social vulnerability and healthcare self-efficacy are inversely related. The moderately strong magnitude justifies combining the latent variables in a single latent variable in the MIMIC model as per the conceptual model.

The final MIMIC model is shown in Fig. 4. The estimated weights, loadings and goodness-of-fit statistics are calculated with the disturbance (error) term. The single latent variable is presumed to be “Social vulnerability to low healthcare self-efficacy”.

### Representing the social vulnerability index

Both PLS-PM and MIMIC express social vulnerability as a weighted sum of the formative indicators. However, the model-generated weight estimates are specific to the model specification and to this empirical dataset [33]. For instance, the model-generated Social Vulnerability Index for our final PLS-PM model would be: Index = (0.42 × Education status) + (0.38 × Financial status) + (0.33 X Instrumental support) + (0.51 × Social support), which is not very pragmatic for clinical practice. And since a different data set would generate slightly different weights, it is not generalizable.

To make the Social Vulnerability Index both generalizable and pragmatic, we transformed weights by assigning a fixed base weight value of 1 to model-generated weights of approximately similar value (e.g. ≈0.4 = 1). In both PLS-PM and MIMIC the weight of the indicators are approximately equivalent, supporting a fixed weight = 1. The result is that characteristics above the vulnerability cut-off can be summed easily to represent total social vulnerability. The proposed Social Vulnerability Index to low healthcare self-efficacy becomes = (1 × Education status) + (1 × Financial status) + (1 × Instrumental social support) + (1 × Confidant social support). Or more simply, the sum of the indicator cut-offs shown in Table 3. The Pearson correlations between the model-generated and the fixed-weight Index is very high: 0.983 with the PLS-PM-generated Index and 0.982 with the MIMIC-generated Index, indicating minimal loss of information with the fixed-weight Index.

### Predicting increased likelihood of negative healthcare events

Our last step was to examine whether and how the Index predicted the likelihood of negative healthcare events in the following 12-month period. We used separate logistic regression models to determine if the Index predicted likelihood of negative healthcare events. Using administrative data we determined if the Index predicted: 2 + visits to the hospital Emergency Department (ED); any hospital admission, and; hospital admissions from the ED (presumed to be unplanned). We used survey data to predict: self-reported use of ED for system-related reasons (not have a family physician or being able to contact primary care, not knowing what to do); unmet needs for care; feeling abandoned in the system, and perception that a health problem became more serious because of delayed care. We repeated the analyses for 2 years after baseline. We examined whether the effect of the index of social vulnerability was independent of or interacted with chronic disease burden.

**Fig. 5:**
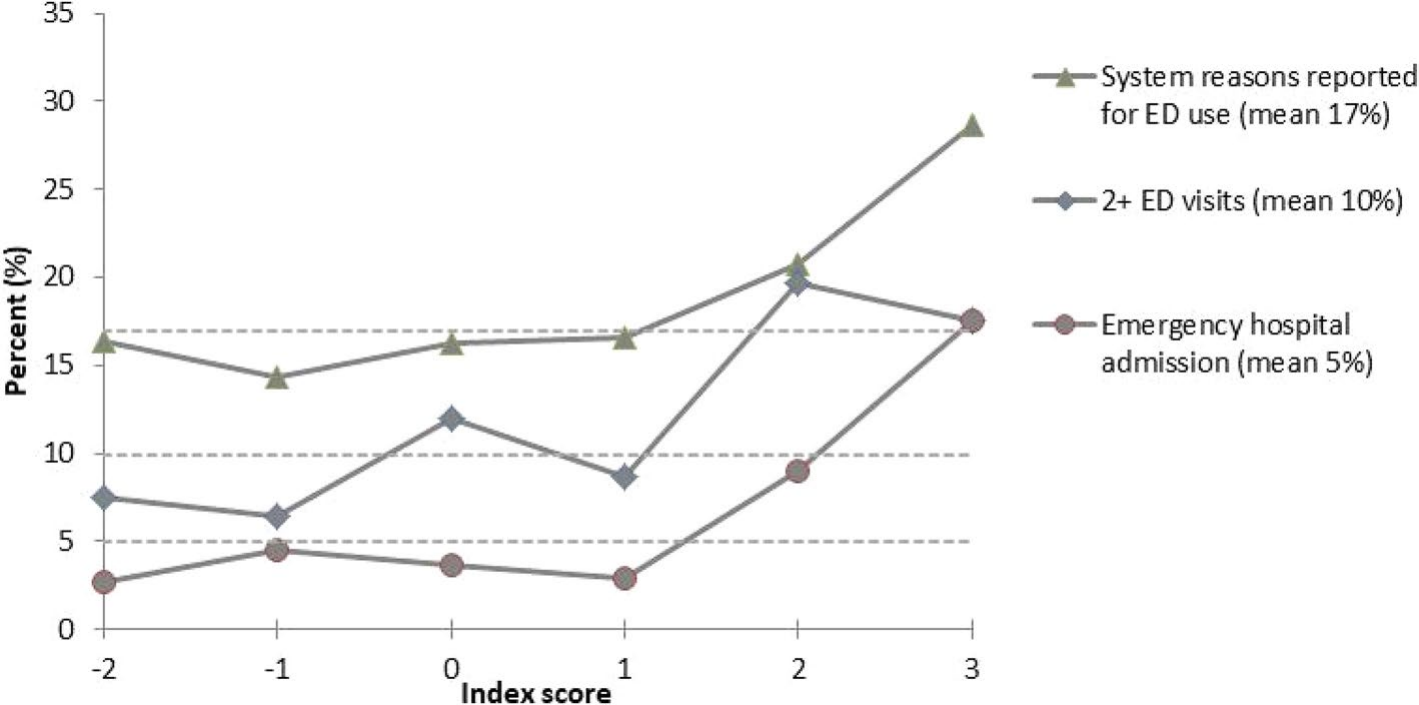
Prevalence of negative healthcare events by Index score compared to average event rate (dotted line)

Graphs of the Index against the occurrence of all the negative healthcare events shows that there appears to be a threshold at Index = 2, where we see an exponential increase in the likelihood of negative healthcare (Fig. 5). In contrast, the relationship of negative healthcare events with chronic disease burden is ordinal, with an increased prevalence of negative events with each additional chronic illness, and no clear threshold. We confirmed with Logistic regression models that Index ≥ 2 predicts an increased likelihood in the subsequent year of all negative healthcare events, except unmet need for care. Consequently, we consider any two of the vulnerability indicators to indicate Social Vulnerability to Poor Healthcare as a binary variable.

When the number of chronic diseases is added to the regression model, the effect of the Social Vulnerability Index ≥ 2 decreases slightly, suggesting confounding by health status, but the effect of social vulnerability remains largely independent of chronic disease burden (Table 4). We explored interaction effects between social vulnerability and chronic disease burden and found a suggestive interaction (*p* = 0.06) for increased risk of hospital admission or feeling abandoned by the health system. In these models the main effect of social vulnerability is not statistically significant, suggesting that in the absence of any chronic disease, social vulnerability alone does not increase risk of hospital admission or feeling abandoned by the health system. However, being socially vulnerable with chronic disease increases exponentially the likelihood of these negative healthcare events relative to the effect of chronic disease alone.

**Table 4:** Summary of logistic regression models with both Index of social vulnerability and number of chronic illnesses included in the model

**Negative healthcare event**	**Prevalence of event**^a^	**Model Results**	
		**Social Vulnerability Index ≥ 2** **OR**^b^ **(95% CI)**	**Number chronic diseases** **OR**^c^ **(95% CI)**
**Events based on administrative data:**			
2 + ED visits (administrative data)	11.1% (197/1769)	**2.18** (1.43, 8.10)	**1.19** (1.09. 1.32)
Any hospital admission	16.3% (289/1769)	**1.50** (1.03. 2.18)	**1.24** (1,15, 1.35)
Hospital admission through the emergency room	4.7% (68/1368)	**2.95** (1,71, 5.07)	**1.38** (1.23, 1.57)
**Self-reported events, survey data**			
ED use for health system reasons^d^	16.9% (343/1685)	**1.61** (1.19, 2.19)	1.04 (0.98, 1.18)
5 + point decline in functional health status (SF-12)^e^	23.1% (414/1377)	1.26 (0.90, 1.75)	**1.32** (1.20, 1.46)
Problem became worse because of delayed care	8.6% (173/1837)	**2.0** (1.36, 2.91)	**1.17** (1.07, 1.27)
Feel abandoned by the system	23.1% (463/1453)	**1.37** (1.03, 1.83)	**1.09** (1.01, 1.15)
Unmet need for healthcare	11.5% (231/1774)	1.16 (0.80, 1,70)	1.08 (0.99, 1.17)

^a^Varying denominator reflects missing values at Time 2

^b^Odds Ratio (OR) show adjusted likelihood of negative healthcare event in the subsequent 12 months, among patients with Index ≥ 2, compared to Index < 2; bolded values are statistically significant two-sided *p* < 0.05

^c^Odds ratio associated with each additional chronic disease from a list of 14 stable diagnoses

^d^Doctor not available, wait for appointment too long, not know what to do, confused what to do or had conflicting information, too far to clinic

^e^Controlling for baseline SF-12

## Discussion

This secondary analysis of cohort data has led to the proposal of an Individual Social Vulnerability Index, a multidimensional construct estimated as the weighted sum of a set of social characteristics. The index is inversely related with a construct of healthcare self-efficacy. The social characteristics are scored to reflect increasing vulnerability, with simplified weights where each vulnerability = 1, which can be summed easily in to the Individual Social Vulnerability Index with higher values corresponding to higher social vulnerability. There is a clear threshold of increased susceptibility to negative healthcare events with Index of Social Vulnerability ≥ 2.predicting increased likelihood of negative healthcare events such as ED use and unplanned hospitalizations. The Individual Social Vulnerability Index shows little confounding between social characteristics and health status, making it appropriate for epidemiologic purposes and to evaluate the inequity or inequality of healthcare delivery. The threshold value makes it useful for clinicians to elicit social characteristics and take action when persons are identified as vulnerable to negative healthcare events. Our goal is to present this as an first demonstration of approach that we believe will be relevant for use in reporting on health system equity and in clinical practice for detecting patients who need more robust support in obtaining appropriate care.

This article tells the story of health services researchers on a quest to find tools that represent and address healthcare inequity and were quickly confronted with the limitation of their toolbox and their knowledge base. Statisticians were able to see viable analytic approaches in depictions of our conceptual dilemmas, and hold us to account to respect the underlying statistical assumptions. Consultations with colleagues ensured that the findings made sense for both research and clinical purposes. We recognized that our common-sense use of ‘vulnerable’ conflated notions of risk, need, assets and capacity. Literature in other domains provided conceptual clarity to understand the Latin *vulnerabilis,* as the susceptibility—*abilis*—to wounding or injury—*vulnus*.

The homeostasis model of vulnerability, proposed Ashby [34, 35] for the domain of cybernetics was particularly influential. It posits that a system or organism maintains stability and resilience by having multiple inter-related and redundant mechanisms for responding to insults. When there are too few overlapping resiliency mechanisms or when mechanisms fail then the capacity of the system to resist or respond is overwhelmed and the system or organism succumbs, even to minor insults. A feature of vulnerability in this model is that system collapse can be sudden and catastrophic, so the goal is to detect and act on risk before collapse. This notion of vulnerability has found resonance in several domains [36–39].

The homeostasis model of vulnerability in the clinical domains is explicit in the development of the Frailty Index [40–43], which has been used extensively in the care of elders. The Frailty Index infers frailty from the proportion of health and functioning deficits in a person. It is ‘blind’ to which specific deficits are present or even how many are elicited in the denominator [23]. What matters is that when a proportional deficit threshold is reached, protective action must be taken to prevent or mitigate negative outcomes. Like the Frailty Index, our Individual Social Vulnerability Index posits that a value of ≥ 2 can be used to flag the minority of patients (approximately 15%) who will need proactive and robust support to avoid health collapse with the concomitant costs and healthcare inequity at a system level.

As with the Frailty Index, we propose that the Individual Social Vulnerability Index be ‘blind’ to which characteristics are at play. This approach is also coherent with the notion of intersectionality, that posits that the co-occurrence of social challenges creates vulnerability that is greater than the sum if its parts [44]. The ‘blindness’ of the Index avoids applying relying on single proxies such as race or financial status, a labelling process that itself can be associated with stigmatization and social discrimination. What matters is to take action when a person has two or more distinct social vulnerabilities.

Our Individual Social Vulnerability Index stands in contrast to the Social Vulnerability Index [45, 46], which was designed to identify communities that would have limited capacity to withstand natural disasters. It reflects “hazard of place” vulnerability which is geographically-centered potential exposure to biophysical hazards such as extreme weather events combined with the social coping responses to the hazard [37, 39]. The Index sums the percentile rank of 15 different social census tract variables (socio-economic, household composition, minority status, housing and transportation), with high values indicating greater community vulnerability. Because the SVI is based on census data, it can be used to map vulnerable communities that require additional resources to mitigate the risks from natural disasters allowing appropriate disaster planning and response. Common to both the Individual and community indices is the notion that having information on vulnerability can be used to mitigate susceptibility to wounding. Frameworks for addressing social determinants of health in primary care require information at both the community and individual level [12, 13].

The coherent results from two structural equation modelling methods with different statistical assumptions gives us confidence in the robustness of our result, but there are limitations and further development is needed. Our study sample is based predominantly on healthcare users in a publicly-funded health care system, and those lost to follow-up had higher social vulnerabilities and were less likely to be attached to a family physician, which we believe biases our results toward the null. Our data were not fit-for-purpose. We did not have sufficient data on race or ethnicity to have the statistical power to examine the effect limited language proficiency or immigration status – though these are known indicators [47]. And we are confident in asserting that low social support, poor financial status, and low educational achievement make unique contributions to the multidimensional construct of social vulnerability, other formulations of these indicators may be better adapted for clinical practice. For instance, clinician colleagues find it awkward to ask our financial status question in a clinical encounter, but Brcic and colleagues found that the binary question “Do you have difficulty making ends meet at the end of the month” has excellent sensitivity for detecting patients below the poverty line, and “After paying your monthly bills do you typically have enough money for food” has excellent specificity. The Center for Medicare and Medicaid Services recommends 10 questions to screen for social vulnerability in clinical settings; they include domains such as interpersonal safety, housing and food insecurity and transportation difficulties [13]. We would say: “Just ask. And if you find 2 or more social vulnerabilities, do something”.

Despite the limitations of our data and study sample, we believe that the use of two complementary structural equation modelling approaches generated a more appropriate measure of this complex multidimensional construct than most indices and measures of social vulnerability or socio-economic status [20, 23, 48–50]. We present this as an initial development, and we look to the community of health and social researchers to take the next steps in testing more sensitive indicators and applying it to different populations. In choosing indicators, we still recommend looking for parsimonious candidate set of the most influential characteristics. The statistical model requires that each candidate characteristic contribute uniquely and not redundantly to the construct of vulnerability. We promote the pragmatic use of the Individual Social Vulnerability Index to detect those needing additional support. In keeping with the homeostatic model a patient with an Index ≥ 2 raises a flag requiring additional support navigating the system, written instructions, easy-to-read health and information, and engagement of available social supports. The easy questions can also trigger more detailed exploration of a wide range of social needs using other available tools, that take longer to administer but can guide specific action [51]. Further work is needed to identify the kinds of support that can be mobilized in primary care to support patients with social vulnerability. Our hope is that the simple and pragmatic approach presented here will make social vulnerability screening a standard of practice while the larger community decides on the best approaches for assessing social determinants of health.

## Conclusion

We privileged parsimony over explanatory capacity in our analytic decisions to make the Individual Social Vulnerability Index pragmatic for epidemiologic and clinical use. The Index can be readily calculated using personal characteristics available in surveys or gathered in clinical practice. The Index demonstrates a threshold effect, where the risk of negative healthcare events increases exponentially at Index ≥ 2. The increased risk of negative healthcare events attributable to social characteristics over and above the risk attributable to poor health makes the Index useful as a multidimensional stratifier for assessing healthcare inequity, but we believe that it is most relevant clinically for identifying individual patients who will who need additional support navigating the system or managing their care in order to avoid health or healthcare collapse.

## Data Availability

Full data reports of quantitative analyses are available from the corresponding author, and the data set can be made available.

## Abbreviations

PCA: Principal Component Analysis
FA: Factor Analysis
ED: Emergency Department
SEM: Structural Equation Modelling
MIMIC: Multiple Indicators Multiple Causes
CFI: Comparative Fit Index
PLS-PM: Partial Least Squares Path Modelling
SD: Standard Deviation

## References

[1] WHO Commission on Social Determinants of Health, World Health Organization. Closing the gap in a generation: health equity through action on the social determinants of health: final report. Geneva: World Health Organization; 2008.

[2] Tudor HJ (1971). The inverse care law. Lancet.

[3] Katz A, Chateau D, Enns JE (2018). Association of the social determinants of health with quality of primary care. Ann Fam Med.

[4] Martin P, Liaw W, Bazemore A, Jetty A, Petterson S, Kushel M (2019). Adults with housing insecurity have worse access to primary and preventive care. J Am Board Fam Med.

[5] Lasser KE, Himmelstein DU, Woolhandler S (2006). Access to care, health status, and health disparities in the United States and Canada: results of a cross-national population-based survey. Am J Public Health.

[6] Aday LA. At risk in America: The health and health care needs of vulnerable populations in the United States, Second Edition. San Francisco: Jossey-Bass Publishers; 2002.

[7] Dahrouge S, Hogg W, Ward N (2013). Delivery of primary health care to persons who are socio-economically disadvantaged: does the organizational delivery model matter?. BMC Health Serv Res.

[8] Levesque J-F, Harris MF, Russell G (2013). Patient-centred access to health care: conceptualising access at the interface of health systems and populations. Int J Equity Health.

[9] Vikum E, Johnsen R, Krokstad S (2013). Social inequalities in patient experiences with general practice and in access to specialists: the population-based HUNT Study. BMC Health Serv Res.

[10] Picot GMJ (2005). Income Inequality and Low Income in Canada: An International Perspective.

[11] Canadian Institute for Heatlh Information C. Resources for measuring health inequalities. 2021. https://www.cihi.ca/en/resources-for-measuring-health-inequalities (Accessed Nov 2022 2022).

[12] DeVoe JE, Bazemore AW, Cottrell EK (2016). Perspectives in primary care: a conceptual framework and path for integrating social determinants of health into primary care practice. Ann Fam Med.

[13] Billioux A, Verlander K, Anthony S, Alley D (2017). Standardized Screening for Health-Related Social Needs in Clinical Settings: The Accountable Health Communities Screening Tool. NAM Perspectives.

[14] Wong S, Haggerty J, Hogg W, et al. Transforming community based primary health care delivery through comprehensive performance measurement and reporting. Vancouver: University of British Columbia Centre for Health Services and Policy Research; 2013.

[15] Haggerty J, Fortin M, Beaulieu M-D (2010). At the interface of community and healthcare systems: a longitudinal cohort study on evolving health and the impact of primary healthcare from the patient's perspectiv. BMC Health Serv Res.

[16] Bayliss E, Ellis J, Steiner J (2005). Subjective assessments of comorbidity correlate with quality of life health outcomes: Initial validation of a comorbidity assessment instrument. Health Qual Life Outcomes.

[17] Ware JEJ, Kosinski M, Keller SD (1996). A 12-item short-form health survey: construction of scales and preliminary tests of reliability and validity. Med Care.

[18] MacCallum RC, Browne MW (1993). The use of causal indicators in covariance structure models: some practical issues. Psychol Bull.

[19] Salmond C, Crampton P (2012). Measuring socioeconomic position in New Zealand. J Prim Health Care.

[20] Salmond C, Crampton P, King P, Waldegrave C (2006). NZiDep: a New Zealand index of socioeconomic deprivation for individuals. Soc Sci Med.

[21] Labbe E, Blanquet M, Gerbaud L (2015). A new reliable index to measure individual deprivation: the EPICES score. Eur J Public Health.

[22] Eroğlu S (2007). Developing an index of deprivation which integrates objective and subjective dimensions: extending the work of Townsend, Mack and Lansley, and Halleröd. Soc Indic Res.

[23] Opatowski M, Blondel B, Khoshnood B, Saurel-Cubizolles M-J (2016). New index of social deprivation during pregnancy: results from a national study in France. BMJ Open.

[24] Wold H (1975). Soft Modelling by Latent Variables: The Non-Linear Iterative Partial Least Squares (NIPALS) Approach. J Appl Probab.

[25] Haenlein M, Kaplan AM (2004). A beginner's guide to partial least squares analysis. Underst Stat.

[26] Wold H. Estimation of principal components and related models by iterative least squares. In: P. R. Krishnajah (Ed.), Multivariate analysis (pp. 391-420). NewYork: Academic Press; 1966.

[27] Lohmöller J-B (1989). Predictive vs. Structural Modeling: PLS vs. ML. Latent Variable Path Modeling with Partial Least Squares.

[28] Hauser RM, Goldberger AS (1971). The treatment of unobservable variables in path analysis. Sociol Methodol.

[29] Jöreskog KG, Goldberger AS (1975). Estimation of a model with multiple indicators and multiple causes of a single latent variable. J Am Stat Assoc.

[30] Jöreskog KG (1978). Structural analysis of covariance and correlation matrices. Psychometrika.

[31] Joreskog KG, Sorbom D (1996). LISREL 8: User's Reference Guide.

[32] Muthén L, Muthén B (2015). Mplus. The comprehensive modelling program for applied researchers: user’s guide.

[33] Hardin AM, Chang JC-J, Fuller MA, Torkzadeh G (2011). Formative measurement and academic research: in search of measurement theory. Educ Psychol Measure.

[34] Ashby WR. An introduction to cybernetics: Chapman & Hall Ltd; 1961.

[35] Ashby WR. Requisite variety and its implications for the control of complex systems. In: Facets of Systems Science. International Federation for Systems Research International Series on Systems Science and Engineering, vol 7. Boston: Springer; 1991. p. 405–17.

[36] Yelin D, Wirtheim E, Vetter P (2020). Long-term consequences of COVID-19: research needs. Lancet Infect Dis.

[37] Mustafa D, Ahmed S, Saroch E, Bell H (2011). Pinning down vulnerability: from narratives to numbers. Disasters.

[38] Mechanic D, Tanner J (2007). Vulnerable people, groups, and populations: societal view. Health Affairs.

[39] Cutter SL (1996). Vulnerability to environmental hazards. Prog Hum Geogr.

[40] Mitnitski AB, Mogilner AJ, Rockwood K (2001). Accumulation of deficits as a proxy measure of aging. Scientific World Journal.

[41] Rockwood K, Andrew M, Mitnitski A (2007). A comparison of two approaches to measuring frailty in elderly people. J Gerontol A Biol Sci Med Sci.

[42] Rockwood K, Mitnitski A (2007). Frailty in relation to the accumulation of deficits. J Gerontol A Biol Sci Med Sci.

[43] Clegg A, Young J, Iliffe S, Rikkert MO, Rockwood K (2013). Frailty in elderly people. The Lancet.

[44] Crenshaw K (1990). Mapping the margins: Intersectionality, identity politics, and violence against women of color. Stanford Law Review.

[45] Cutter S, Boruff B, Shirley W. Social vulnerability to environmental hazards. Soc Sci Q. 2003;84:242–61.

[46] Flanagan BE, Gregory EW, Hallisey EJ, Heitgerd JL, Lewis B. A social vulnerability index for disaster management. J Homeland Security Emerg Manage. 2011;8(1):1–22.

[47] Andrew MK, Mitnitski AB, Rockwood K (2008). Social vulnerability, frailty and mortality in elderly people. PLoS ONE [Electronic Resource].

[48] Vaucher P, Bischoff T, Diserens E-A (2012). Detecting and measuring deprivation in primary care: development, reliability and validity of a self-reported questionnaire: the DiPCare-Q. BMJ Open.

[49] Booysen F, van der Berg S, Burger R, Maltitz MV, Rand GD (2008). Using an asset index to assess trends in poverty in seven Sub-Saharan African countries. World Development.

[50] Sass C, Moulin J-J, Guéguen R (2006). Le score Epices: un score individuel de précarité. Construction du score et mesure des relations avec des données de santé, dans une population de 197 389 personnes. Bulletin Épidémiologique Hebdomadaire.

[51] Moen M, Storr C, German D, Friedmann E, Johantgen AM (2020). A review of tools to screen for social determinants of health in the united states: a practice brief. Popul Health Manage.

